# Tracheal Aspirate Galactomannan Testing in COVID-19-Associated Pulmonary Aspergillosis

**DOI:** 10.3389/ffunb.2022.855914

**Published:** 2022-04-08

**Authors:** Carla M. Román-Montes, Saúl Bojorges-Aguilar, Paulette Díaz-Lomelí, Axel Cervantes-Sánchez, Andrea Rangel-Cordero, Areli Martínez-Gamboa, José Sifuentes-Osornio, Alfredo Ponce-de-León, Maria F. González-Lara

**Affiliations:** ^1^Clinical Microbiology Laboratory, Instituto Nacional de Ciencias Médicas y Nutrición “Salvador Zubirán”, Mexico City, Mexico; ^2^Medicine Direction, Instituto Nacional de Ciencias Médicas y Nutrición “Salvador Zubirán”, Mexico City, Mexico; ^3^Infectious Diseases Department of Medicine, Instituto Nacional de Ciencias Médicas y Nutrición “Salvador Zubirán”, Mexico City, Mexico

**Keywords:** COVID-19, CAPA, *Aspergillus*, galactomannan, tracheal aspirate

## Abstract

Among critically ill patients, COVID-19-associated pulmonary aspergillosis (CAPA) is a challenging complication. The recommended diagnostic methods for this disease are bronchoalveolar lavage (BAL) culture and galactomannan (GM) testing, which were not widely available during the pandemic. There is scarce information regarding GM testing in other respiratory specimens. Our objective was to compare the agreement of GM between BAL and tracheal aspirate (TA) samples. We selected patients with COVID-19 and those with suspected CAPA who were admitted in the intensive care unit (ICU). GM was routinely done in BAL. We performed GM in TA samples and compared the results. The agreement was evaluated with Cohen's Kappa coefficient. GM was considered positive when an OD index ≥ 1 in BAL and ≥ 2 in TA were found. Probable CAPA was considered when the ECMM/ISHAM criteria were met. A descriptive analysis of clinical characteristics and mortality was made. We included 20 patients with suspected CAPA from 54 patients with critical COVID-19, of which 5 (9%) met the probable category. *Aspergillus fumigatus* was the most frequent isolate. We found moderate agreement between BAL and TA GM (Kappa = 0.47, *p* = 0.01, 95% CI.04–0.9), whereas TA GM had 75% sensitivity (95% CI 19.4–99.4%), 81.2% specificity (95% CI 54.4–95.9%), 50% positive predictive value (95% CI 23.8–76.3%),] and 92.8% negative predictive value (95% CI 70.1–98.6%), and 80% accuracy (95% CI 56.3–94.3%). Lastly, three (60%) patients with CAPA died during hospitalization compared to 40% (6/15) without CAPA (*p* = 0.4). In conclusion, a moderate agreement between TA GM and BAL was found. Therefore, TA testing may aid in ruling out CAPA due to high negative predictive value when bronchoscopies are unavailable.

## Introduction

The emergence of the COVID-19 pandemic is a major strain for healthcare systems. Up to 25% of hospitalized patients may need intensive care unit (ICU) care, of which 67% may develop acute respiratory distress syndrome (ARDS) (Tzotzos et al., 2020; Yang et al., 2020). In this setting, SARS-CoV-2 infection results in a pro-inflammatory state with immune dysregulation leading to ARDS, organ failure, and coagulopathy. The cytokine storm can lead to T-cell exhaustion with decreased levels of cluster of differentiation 4 (CD4)-lymphocytes contributing to fungal and other superinfection development (Amin et al., 2021). Currently, the standard of care includes dexamethasone in hospitalized patients and interleukin 6 (IL-6) receptor inhibitors in those with increasing oxygen needs and inflammation. Although their role in fungal infection pathogenesis is unclear, a combination of hypoxia, steroid- and stress-induced hyperglycemia, prolonged mechanical ventilation, and COVID-19-associated immune dysregulation affecting T-helper (Th)2 and Th1 responses contribute to increased fungal superinfection (Amin et al., 2021; Feys et al., 2021; National Institutes of Health, 2021; Noreen et al., 2021).

Among critically ill patients in ICU, COVID-19-associated pulmonary aspergillosis (CAPA) is a challenging complication (Prattes et al., 2021b). Although the prevalence of CAPA is variable among series due to different definitions, a global estimate of 10.2% was described in a recent systematic review and meta-analysis (Mitaka et al., 2021; Vélez Pintado et al., 2021). Increased mortality among patients with CAPA compared to other critically ill patients with the SARS-CoV-2 infection has been described (Singh et al., 2021; Chong et al., 2022).

Early in 2021, a consensus to define and improve the diagnosis and treatment of CAPA was published. These recommendations support the use of *Aspergillus* biomarkers such as galactomannan (GM) and PCR in bronchoalveolar lavage (BAL) samples as mycological criteria for probable CAPA (Koehler et al., 2021a). During the pandemic, clinicians faced reluctance to perform aerosol-generating diagnostic procedures such as bronchoscopy, which led to limited availability of BAL samples to perform GM testing. Limited studies explore the use of *Aspergillus* biomarkers in other widely available respiratory specimens, such as tracheal aspirate (TA). Previously, we conducted a study to evaluate GM testing in TA and found that a two optical density (OD) cut-off point had the best area under the curve (AUC), but we did not compare these results with BAL GM (Roman-Montes et al., 2021).

In this study, we aimed to evaluate the agreement, sensitivity, specificity, and predictive values of TA GM compared to BAL samples.

## Methods

### Study Design and Population

Between April and August 2021, we included patients with severe COVID-19 along with suspected invasive fungal infection (IFI) who had available samples of BAL and TA taken within < 7 days. BAL GM was performed routinely, and the result was immediately informed to the treating physicians. TA GM was performed for this study and interpreted by independent microbiologists who were blinded to clinical data and to BAL GM. A case was considered CAPA when the European Confederation of Medical Mycology and the International Society for Human and Animal Mycology (ECMM/ISHAM) probable category was met (Koehler et al., 2021a). Having CAPA means having one of the following: ≥0.5 serum GM or ≥ 1 BAL GM or a positive BAL culture with *Aspergillus* species and the presence of new infiltrates, consolidations, nodules, or cavitary lung lesion(s) on chest CT without alternative explanation. Patients were not considered CAPA when alternative diagnoses were made and the ECMM/ISHAM probable CAPA category was not met. Also, we compared this definition with the European Organization for Research and Treatment of Cancer/Invasive Fungal Infections Cooperative Group and the National Institute of Allergy and Infectious Diseases Mycoses Study Group (EORTC/MSG) and modified the AspICU criteria (Schauwvlieghe et al., 2018; Bassetti et al., 2021).

### Laboratory Procedures

BAL and TA samples were routinely processed for bacterial and fungal cultures, and gram and potassium hydroxide (KOH) stains were performed directly on arrival to the Clinical Microbiology Laboratory. TA samples were not treated with mucolytics. GM detection was performed in 300 μL of BAL and AT samples using the Platelia™ Aspergillus Ag kit, BioRad Lab®, Redmond, WA, USA. following the manufacturer's recommendations. The interpretation of positive GM was as follows: in BAL with an OD index ≥ 1.0, in TA with an OD ≥ 2.0 index (Roman-Montes, C. M., 2021) and in serum sample with GM index >0.5 (Roman-Montes et al., 2021).

### Statistical Analysis

We used descriptive and comparative analyses to describe the characteristics of patients with and without CAPA through X2 test, T-test, Mann-Whitney U test or exact Fisher test as appropriate. We used Cohen's Kappa test to evaluate the agreement between TA GM and BAL GM results. We calculated sensitivity, specificity, and positive and negative predictive values. An AUC model was done. We used Stata 12 Statistics software®, Texas, USA.

### Ethics

Written informed consent was waived due to the observational nature of the study, and the procedures performed were part of the standard care of the patient. We kept the anonymity of the patients. The study did not influence the treatment clinician's decision. The study was approved by the Institutional Review Board (Local Reference number 3384).

## Results

We included 20 critically ill patients with suspected CAPA from 54 patients with severe COVID-19 between April and August 2021. Demographic and clinical characteristics are described in Table 1. Only five patients (9%) met the criteria for probable CAPA. One patient with *Aspergillus fumigatus* growth in TA was considered colonization. None had neutropenia or met the EORTC/MSG criteria, whereas all met the modified AspICU criteria.

**Table 1:** Clinical characteristics of critically ill patients with COVID-19 along with probable COVID-19-associated pulmonary aspergillosis (CAPA).

**Characteristics**	**All *N =* 20 (%)**	**Probable CAPA** ***N* = 5 (25%)**	**Not CAPA** ***N* = 15 (75%)**	***p*-value**
Age, median (IQR)	55 (41.5–67)	53 (43–55)	56 (40–68)	0.51
Male sex	17 (85)	5 (100)	12 (80)	0.27
Obesity	13 (65)	2 (40)	11 (73)	0.17
Hypertension	13 (65)	2 (33)	11 (78.5)	0.01
Diabetes	7 (35)	1 (20)	6 (40)	0.41
**COVID-19 treatment**				
Corticosteroids	19 (95)	4 (80)	15 (100)	0.07
Remdesivir	4 (20)	1 (20)	3 (20)	0.27
SOFA score, mean (SD)	6 (2.2)	5.2 (1.5)	6 (2.4)	0.46
APACHE II, mean (SD)	10 (3.5)	11 (2)	10 (4)	0.6
Bacterial coinfection	15 (75)	4 (80)	11 (73)	0.76
Antifungal treatment	11 (55)	5 (100)	6 (40)	0.12
ICU stay length, mdn days (IQR)	31.5 (21–48)	21 (12–41)	33 (22–52)	0.18
Hospital length stay, mdn days (IQR)	46 (26–63.5)	22 (12–51)	48 (41–66)	0.17
Death	9 (45)	3 (60)	6 (40)	0.43

*CAPA, COVID-19-associated pulmonary aspergillosis; ICU, intensive care unit; SD, standard deviation; mdn, median*.

*Obesity: BMI ≥30 kg/m2. Hypertension was considered when systolic blood pressure levels ≥ 130 mmHg or diastolic blood pressure levels ≥ 80 mmHg*.

Among patients with CAPA, all of them were men with a median age of 53 (IQR 43–55) years, with a body mass index (BMI) of 30.39 (IQR 26.6–32.6) kg/m2, among which two (40%) had obesity, two (40%) had hypertension, and one had Type 2 diabetes mellitus. The mean Sequential Organ Failure Assessment (SOFA) score was 5.2 (*SD* 1.5) and the mean Acute Physiology and Chronic Health Evaluation 2 (APACHE II) score was 11 (*SD* 2). All baseline characteristics were similar between patients with and without CAPA, except for fewer corticosteroid treatments in patients with CAPA (80% vs 100%, RR 0.21, 95% CI 0.08–0.5, *p* = 0.07). All patients with CAPA had new infiltrates in CT, four had consolidations, two had consolidations and ground-glass opacities, and 1 with pulmonary thrombosis, but none had cavitation. Among 15 patients without CAPA, all had new ground glass infiltrates in CT.

Furthermore, four patients with CAPA (80%) received antifungal treatment with voriconazole, while six patients without CAPA (40%) received empirical antifungal treatment due to high IFI suspicion (4 anidulafungin and 2 combined voriconazole with anidulafungin). A shorter non-significant median hospital stay [22 days (IQR 12–51)] and ICU stay length [21 days (IQR 12–41)] were seen in patients with CAPA. Also, patients with CAPA died earlier during ICU stay. Lastly, three (60%) patients with CAPA died during hospitalization compared to six (40%) out of 15 patients without CAPA (OR 2.25, 95% CI.28–17.7, *p* = 0.44) (Table 1).

Microbiological characteristics of CAPA patients are described in Table 2. Serum GM was negative in one, while BAL GM was positive in three, of which one was culture-negative, one developed *Aspergillus niger* in BAL culture, and two grew *A. fumigatus* in both TA and BAL samples. Sixty percent (3/5) had a bacterial co-infection (Table 2). Among patients without CAPA, 12 had positive bacterial cultures, while 3 had negative BAL and TA cultures and were considered COVID-19 progression.

**Table 2:** Clinical and microbiological characteristics of patients with CAPA.

	**Case 1**	**Case 2**	**Case 3**	**Case 4**	**Case 5**
Sex	Male	Male	Male	Male	Male
Age	55	24	53	43	86
Comorbidities	Obesity	Systemic lupus erythematosus Antiphospholipid syndrome	Hypothyroidism	Obesity Hypertension Diabetes type 2 Ischemic heart disease	Renal transplant Treatment with prednisone 5mg
COVID-19 treatment	Steroid	Remdesivir Steroid	Steroid	None	Steroid
CAPA Diagnosis: fungal cultures	**BAL culture** *A. fumigatus*	**BAL culture**: negative	**BAL culture**: negative	**BAL culture**: *A. niger*	**BAL culture**: *A. fumigatus*
	**TA culture**: *A. fumigatus*	**TA culture**: negative	**TA culture**: negative	**TA culture**: negative	**TA culture**: *A. fumigatus*
CAPA Diagnosis: galactomannan	**BAL**: positive (OD 6.1)	**BAL**: positive (OD 1.43)	**BAL**: positive (OD 1.0)	**BAL:** negative (OD 0.48)	**BAL:** positive (OD 10.5)
	**TA**: positive (OD 10.0)	**TA**: negative (OD 0.09)	**TA**: positive (OD 4.2)	**TA:** positive (OD 2.0)	**TA:** positive (OD 10.5)
					**Serum:** Negative
CT finding	Consolidation	Consolidation	Consolidation and ground glass opacities	Consolidation and ground glass opacities	Ground glass opacities and thrombosis
Bacterial coinfection	**BAL** and **TA**: None	**BAL**: *S maltophilia, Pantoea agglomerans*	**BAL:** *S. marcescens*.	**BAL:** no isolation	**BAL:** no isolation
		**TA**: *Enterobacter Cloacae, S. maltophilia*	**TA:** *K. pneumoniae, S. marcescens*	**AT:** *E. coli*	**AT:** yeast isolation
Antifungal treatment	Voriconazole switch to anidulafungin	Voriconazole	Voriconazole	Voriconazole	Empirical anidulafungin, then switch to Voriconazole
Outcome (after CAPA diagnosis)	Died (8 days)	Hospital discharge (47 days)	Hospital discharge (40 days)	Died (4 days)	Died (5 days)

*BAL, Bronchoalveolar lavage; TA, tracheal aspirate; CAPA, COVID-19-associated pulmonary aspergillosis; CT, computed tomography; ICU, intensive care unit*.

The median time between BAL and TA sampling was 1 day (Range: 0–4). Agreement between BAL and TA GM was 80% with moderate concordance (Kappa = 0.47, *p* = 0.01, 95% CI.04–0.9). TA GM had 75% sensitivity (95% CI 19.4–99.4%), 81.2% specificity (95% CI 54.4–95.9%), 50% positive predictive value (95% CI 23.8–76.3%) and 92.8% negative predictive value (95% CI 70.1–98.6%), and 80% accuracy (95% CI 56.3- 94.3%) for CAPA diagnosis.

Among discordant BAL and TA GM results, three samples had positive TA GM (OD 2, 8.5, and 3.8) and negative BAL GM, of which one had a BAL culture with *A. niger* and was considered probable CAPA and the other two had *Klebsiella pneumoniae* and *Pseudomonas aeruginosa* in TA culture, respectively, and were not considered CAPA. Also, one patient with a positive BAL GM (OD 1.4), a negative TA GM (OD.09), and *Stenotrophomonas maltophilia* growth on both cultures were considered probable CAPA.

## Discussion

In this study of patients with severe COVID-19 along with suspected CAPA, we found moderate agreement between TA and BAL GM. Moderate sensitivity (75%), specificity (81%), and a high (93%) negative predictive value of TA GM could aid the clinical decision to rule out CAPA in settings with low resources or limited access to bronchoscopies.

Early in the pandemic, the use of bronchoscopies among patients with severe SARS-CoV-2 infection was discouraged because of aerosol generation concerns, resulting in a high occupational risk among healthcare workers (Wahidi et al., 2020). Also, frequent circumstances affecting critically ill patients with ARDS, such as hemodynamic instability, high positive end-expiratory pressure (PEEP), and coagulopathy, may preclude bronchoscopy, rendering TA culture or non-directed bronchial lavage (ND-BL) as the only available respiratory samples (Mahmoud et al., 2022).

However, testing biomarkers such as GM or PCR has not been widely validated in other types of respiratory samples, resulting in uncertainty about their utility. Recently, a study from the Netherlands described a systematic screening strategy for CAPA diagnosis and found 86% agreement between TA and BAL *Aspergillus* PCR in ventilated patients with COVID-19. In this pilot study, 24% had a positive screening test, and probable CAPA was ultimately diagnosed in 17%. These results are encouraging for TA PCR testing with an overall 86% concordance among 22 samples who had both TA and BAL. In this study, GM was performed in 10 random samples, and 3 weakly positive results were found (OD range 0.51–1.36). Unfortunately, these results were neither directly compared to a simultaneous BAL GM nor interpreted with a more suitable breakpoint for this type of sample (Grootveld et al., 2021).

Another diagnostic strategy includes ND-BL testing, which was introduced when White et al., conducted a screening strategy in ICU patients with refractory or respiratory function worsening at 1-week after COVID diagnosis. They performed GM and PCR on serum and ND-BL samples and were able to detect 26.7% CAPA incidence. A GM positivity threshold of ≥ 1 for ND-BL was used, but a direct comparison between ND-BL and BAL samples was not made. Although ND-BL is less validated, increased mortality among untreated patients with positive ND-BL compared to those who received antifungal treatment was seen (46 vs. 100%) (White et al., 2021). Also, Van Biesen et al., performed ND-BL *via* the closed-circuit suction catheter to identify ICU patients with suspected CAPA and found 21.4% Putative Invasive Pulmonary *Aspergillosis*. In this study, a GM cutoff of the OD index of ≥1 was used, and 77% positive GM tests ultimately yielded positive *Aspergillus* cultures (Van Biesen et al., 2020). These results show respiratory samples other than BAL can identify patients who benefit from treatment. Further evaluation of a suitable GM cutoff value in this type of sample is needed.

Several risk factors have been described for CAPA, such as chronic lung diseases, intubation and mechanical ventilation, corticosteroid treatment, and the cytokine storm in severe SARS-CoV-2 infection (Gangneux et al., 2020). In a large multicenter study including 565 mechanically ventilated patients with COVID-19, 15% met the criteria for proven or probable CAPA. An age older than 62 years, treatment with the combination of dexamethasone and IL-6 antagonists, and prolonged mechanical ventilation duration were independently associated with CAPA (Gangneux et al., 2022).

In our study, we found a 9% CAPA prevalence among ventilated patients with COVID-19, similar to the global estimate (Mitaka et al., 2021). Distinct series show a wide variability (3–34%) in the incidence, due to different diagnostic criteria and definitions. In a study from Spain, a 3% prevalence was found using the strict EORTC/MSG criteria in immunosuppressed patients and the modified AspICU criteria in the rest (Machado et al., 2021). In contrast, a prospective study from Germany included 32 critically ill patients with COVID-19 and found a 34% CAPA prevalence. In this study, using GM in ND-BAL samples, probable CAPA cases were classified with less strict modified AspICU/CAPA criteria, which may explain a higher prevalence (Lahmer et al., 2021). One report from Mexico found a 16% prevalence of probable cases using CAPA criteria, wherein they included only those who have requested GM, which may overestimate the prevalence. There was a higher frequency of cancer but a lesser use of steroids and mortality (Vélez Pintado et al., 2021).

Our study has some limitations, reduced sample size and the number of BAL samples for direct comparison, mainly because bronchoscopies were performed after April 2021 in our institution. Early on in the pandemic, concern for high-risk aerosol-generating procedures increasing the risk of infection among healthcare workers and hospital overburden precluded this procedure. Further, despite accumulating knowledge regarding how adequate use of personal protective equipment and safety strategies can minimize contamination during bronchoscopy, the high-risk status of patients with severe ARDS resulting in hemodynamic instability or prone positioning resulted in few patients who could undergo bronchoscopies. TA sampling could overcome several disadvantages of more invasive procedures (Guillon et al., 2018; Koehler et al., 2021b; Mehta et al., 2021; Bansal et al., 2022). Another limitation was the lack of available reports of bronchoscopy findings in several cases, which may lead to underestimation of COVID-19-associated tracheobronchitis.

We could not compare our index test (TA GM) with the gold standard for invasive fungal infection (sterile cultures or histopathology: proven CAPA cases) because no patient was subject to lung biopsy or autopsy. This is a frequent limitation when assessing the pathogenesis of CAPA since proven cases are anecdotal among large registries (1.9%) (Chong and Neu, 2021). Furthermore, the rate of autopsies during the pandemic is low, and novel techniques such as ultrasound-guided minimally invasive autopsy and postmortem computed tomography may help clinicians to diagnose COVID-19 complications or characteristics (Fitzek et al., 2021). Also, many cases of CAPA or tracheobronchitis do not have evidence of angioinvasion (Flikweert et al., 2020; van de Veerdonk et al., 2021). In our study, serum GM was available for a single case. However, poor performance has been described in the setting of CAPA in contrast to influenza-associated pulmonary aspergillosis or invasive aspergillosis in immunocompromised hosts (Verweij et al., 2020). In this setting, evaluating new diagnostic biomarkers in respiratory samples can only be compared to GM BAL testing and the probable CAPA category (Prattes et al., 2021a).

A major strength of the study is the prospective diagnostic study design that includes selecting consecutive patients with suspected CAPA, reducing the spectrum bias. Also, a blind interpretation of TA from BAL GM and clinical data reduces the incorporation of the bias risk (Kea et al., 2019).

In this pilot study, we found that TA GM had an elevated negative predictive value, which may allow us to rule out CAPA as the cause of respiratory insufficiency worsening. We believe that these data may provide useful information during decision making, particularly in settings where there are no widely available bronchoscopies or trained personnel, as it happened in overburdened health systems during the pandemic, or where patients with severe ARDS may preclude this kind of sampling. Also, negative TA GM allows the suspension of empirical antifungal treatment, allowing better antimicrobial stewardship. Large, prospective studies that further validate our findings are needed.

## Conclusion

Tracheal aspirate GM had a moderate concordance with BAL GM in a small sample of patients with CAPA. A high negative predictive value may allow discarding this diagnosis when BAL is not available.

## Data Availability Statement

The raw data supporting the conclusions of this article will be made available by the authors, without undue reservation.

## Ethics Statement

The studies involving human participants were reviewed and approved by Comité de Investigación en Humanos, Instituto Nacional de Ciencias Medicas y Nutricion Salvador Zubiran. Written informed consent for participation was not required for this study in accordance with the national legislation and the institutional requirements.

## Author Contributions

CR-M, SB-A, AM-G, JS-O, AP-d-L, and MG-L designed the study. CR-M, SB, and PD-L captured and analyzed the data. AR-C, PD-L, and AC-S determined GM in samples and laboratory procedures. All authors contributed to writing the manuscript.

## Funding

This research was supported by Microbiology Laboratory Research funding.

## Conflict of Interest

The authors declare that the research was conducted in the absence of any commercial or financial relationships that could be construed as a potential conflict of interest.

## Publisher's Note

All claims expressed in this article are solely those of the authors and do not necessarily represent those of their affiliated organizations, or those of the publisher, the editors and the reviewers. Any product that may be evaluated in this article, or claim that may be made by its manufacturer, is not guaranteed or endorsed by the publisher.
